# Facile Fabrication of BiF_3_: Ln (Ln = Gd, Yb, Er)@PVP Nanoparticles for High-Efficiency Computed Tomography Imaging

**DOI:** 10.1186/s11671-021-03591-2

**Published:** 2021-08-14

**Authors:** Jun Xie, Zonglang Zhou, Sihan Ma, Xian Luo, Jiajing Liu, Shengyu Wang, Yuqiang Chen, Jianghua Yan, Fanghong Luo

**Affiliations:** 1 Cancer Research Center, Medical College, Xiamen University, Xiamen, 361102 China; 2 College of Energy, Xiamen University, Xiamen, 361102 China; 3 The 174th Clinical College of People’s Liberation Army, Anhui Medical University, Hefei, 230032 China

**Keywords:** Facile synthesized strategy, BiF_3_: Ln@PVP nanoparticles, Contrast agents, Computed tomography, Gastrointestinal tract imaging

## Abstract

X-ray computed tomography (CT) has been widely used in clinical practice, and contrast agents such as Iohexol are often used to enhance the contrast of CT imaging between normal and diseased tissue. However, such contrast agents can have some toxicity. Thus, new CT contrast agents are urgently needed. Owing to the high atomic number (*Z* = 83), low cost, good biological safety, and great X-ray attenuation property (5.74 cm^2^ kg^−1^ at 100 keV), bismuth has gained great interest from researchers in the field of nano-sized CT contrast agents. Here, we synthesized BiF_3_: Ln@PVP nanoparticles (NPs) with an average particle size of about 380 nm. After coating them with polyvinylpyrrolidone (PVP), the BiF_3_: Ln@PVP NPs possessed good stability and great biocompatibility. Meanwhile, compared with the clinical contrast agent Iohexol, BiF_3_: Ln@PVP NPs showed superior in vitro CT imaging contrast. Subsequently, after in situ injection with BiF_3_: Ln@PVP NPs, the CT value of the tumor site after the injection was significantly higher than that before the injection (the CT value of the pre-injection and post-injection was 48.9 HU and 194.58 HU, respectively). The morphology of the gastrointestinal (GI) tract can be clearly observed over time after oral administration of BiF_3_: Ln@PVP NPs. Finally, the BiF_3_: Ln@PVP NPs were completely discharged from the GI tract of mice within 48 h of oral administration with no obvious damage to the GI tract. In summary, our easily synthesized BiF_3_: Ln@PVP NPs can be used as a potential clinical contrast agent and may have broad application prospects in CT imaging.

## Introduction

X-ray computed tomography (CT) can image internal tissues and organs in a cross-sectional way with high resolution and low price [1, 2]. Thus, it is an important means to diagnose respiratory diseases, digestive diseases, and urinary system diseases [3–8]. However, CT sometimes has low contrast between diseased tissues and normal tissues. Thus, contrast agents such as Iohexol are widely used in clinical practice to specifically enhance the X-ray attenuation of diseased tissues. However, the clinical contrast agents are often used in large doses due to the low sensitivity of CT detectors [9]. In addition, commercial iodine-based contrast agents have an extremely fast metabolism in the body and severe side effects, including cardiac events and nephrotoxicity; these issues limit their clinical use and should be solved urgently [10–15].

Nanomaterials have shown broad application prospects in environmental remediation, photovoltaic applications, catalysts, etc. [16–21]. For instance, Balati et al. [22] have synthesized a heterostructured photocatalyst (HBTiO_2_/RBIHM-MoS_2_) using pulsed laser ablation in liquid (PLAL) followed by microwave irradiation. Nanomaterials have also been widely used in medicine, including imaging and treatment.

Gold (Au), tantalum (Ta), platinum (Pt), and other elements with high X-ray attenuation have gained the interest of researchers, and nanomaterials synthesized from these elements have been well-researched as potential contrast agents for CT imaging [1, 12–15, 23, 24]. However, their high price and uncertain biosafety limits their further use. Bismuth (Bi) is well-known as a biosafe element with low cost. It has been used in clinical practice and plays a critical role in combination therapy for *Helicobacter pylori* and other diseases, including chronic liver disease as well as gastric and duodenal ulcers. It has great biological safety and tolerance during treatment [7, 25]. Also, Bi has been used in the preparation of nanoscale contrast agents such as HA-BiO NPs, Bi_2_S_3_, BION, and Bi_2_Te_3_ because of its high atomic number (*Z* = 83) and excellent X-ray attenuation capacity (5.74 cm^2^ kg^−1^ at 100 keV) [26–29].

For instance, Mohsen Mahvi et al. synthesized Bi_2_Te_3_ nanoflakes via a microwave-assisted polyol process that showed a better X-ray attenuation coefficient than commercial Iohexol [29]. Thus, Bi is a promising element for constructing high-performance CT contrast agents. However, the preparation of Bi-based nanocontrast agents is complicated [30, 31].

Here, we combined Bi with lanthanides (Gd, Yb, Er) via a facile and low-cost protocol to fabricate BiF_3_: Ln@PVP nanoparticles (NPs). We then investigated its potential for generating contrast for CT imaging. After coating the samples with PVP, BiF_3_: Ln@PVP NPs showed good stability and low biological toxicity. These samples exhibit better X-ray attenuation than commercial Iohexol in vitro, have good in vivo contrast, and offer great gastrointestinal (GI) tract CT imaging. Importantly, after 48 h of oral administration of BiF_3_: Ln@PVP, the nanoparticles were completely excreted from the body showing no obvious damage to vital organs such as the liver and the kidneys. We believe that our work may provide a new theoretical basis for the clinical use of nanoscale CT contrast agents.

## Methods

All experimental protocols including animal experiments were approved by the ethics committee of Xiamen University in Fujian Province, China.

### Materials and Reagents

Bismuth nitrate pentahydrate (Bi(NO_3_)_3_·5H_2_O, ≥ 99.99%), ammonium fluoride (NH_4_F, ≥ 99.99%), ytterbium nitrate hexahydrate (Yb(NO_3_)_3_·6H_2_O, ≥ 99.9%), erbium nitrate hexahydrate (Er(NO_3_)_3_·6H_2_O, ≥ 99.9%), gadoliniumnitrate hexahydrate (Gd(NO_3_)_3_·6H_2_O, ≥ 99.9%), polyvinylpyrrolidone (PVP, ≥ 99.0%), and Iohexol (≥ 99.0%) were bought from Aladdin Reagents (Shanghai, China). The live-dead cell staining kit and cell counting kit-8 (CCK-8) were bought from Yeasen (Shanghai, China). The RPMI medium 1640, penicillin, streptomycin, and fetal bovine serum (FBS) were purchased from Gibco (New York, USA).

### Fabrication of BiF_3_: Ln@PVP NPs

The BiF_3_: Ln@PVP NPs were synthesized via a hydrothermal approach. In detail, 1 mmol Ln(NO_3_)_3_, (Ln = Yb, Er and Gd), and 1 mmol Bi(NO_3_)_3_ were dissolved into a 35-mL solution including 5-mL deionized water (DI) and 30-mL ethylene glycol to form a transparent solution A. This was then mixed with 0.5 g PVP (M_W_ = 10,000) and stirred at room temperature for 10 min. NH_4_F (20 mmol) was dissolved into 10-mL DI to form solution B. Solution B was then poured into solution A, and a white mixture solution C was formed after stirring for 20 min. Solution C was then put into a 50-mL autoclave and heated to 180 °C for 24 h. The temperature naturally dropped to room temperature after 24 h. Finally, the samples were centrifuged (8000 rpm, 3 min) and rinsed by DI and alcohol to wash away the un-reacted substances. The last samples were gathered by freeze-drying.

### Characterization of BiF_3_: Ln@PVP NPs

The morphology of BiF_3_: Ln@PVP NPs was detected by transmission electron microscopy (TEM, TECNAI G20 F30 TWIN, Oxford) with an operating voltage of 300 kV. The composition of the nanoparticles was analyzed by energy dispersive spectrum (EDS) in TEM including map analysis. The Fourier transform infrared spectroscopy (FTIR, Thermo Scientific Nicolet iN10 MX spectrometer, USA) was used to distinguish the functional groups of the samples. The crystal structures and phase feature of the BiF_3_: Ln@PVP NPs used powder X-ray diffraction (XRD, D8 Advance) with Cu Kα radiation under 40 kV and 40 mA conditions. The size distribution of the nanoparticles dispersed in DI and PBS (pH 7.4) was investigated by dynamic light scattering (DLS, Brookhaven Instruments-Omni, USA).

Cell Line and Cell Culture: HepG2 cells were from the Cell Bank of the Chinese Academy of Sciences (Shanghai, China). Cells were cultured in RPMI medium 1640 containing 10% fetal bovine serum (FBS) and 1% penicillin-streptomycin under 37 °C and 5% CO_2_ conditions. The culture media was replaced every other day.

### Cytocompatibility of BiF_3_: Ln@PVP NPs In Vitro

The cytocompatibility of BiF_3_: Ln@PVP NPs in vitro was estimated by a live-dead assay and the CCK-8 assay. In detail, the HepG2 cells were collected and seeded in confocal dishes at 5.0 × 10^5^. The cells were then cultured overnight. The BiF_3_: Ln@PVP NP suspensions were next added to the cells at different concentrations (100, 200, and 400 μg/mL) and set as the experimental groups. Meanwhile, medium without nanoparticles was added and set as the control group. Subsequently, both the experimental groups and the control group were cultured for 24 h. After 24 h, we gently removed the original medium and the live-dead assay was then performed according to the protocol provided by the manufacturer. Briefly, the living cells were labeled via Calcein-AM, while the dead cells were stained by propidium iodide (PI); cells were then observed under a confocal microscope (Nikon, Japan).

A CCK-8 assay was performed to further determine the cytotoxicity of BiF_3_: Ln@PVP NPs in vitro. In detail, the HepG2 cells were collected and seeded in a 96-well plate with 3000 cells per well and cultured in an incubator overnight. Different concentrations of BiF_3_: Ln@PVP NP (0, 25, 50, 100, 200, and 400 μg/mL) were mixed with the cells and cultured for 24 h. The CCK-8 reagent (10 μL) was added into each well and incubated for 2 h at 37 °C conditions. Later, the OD values of each well were measured at 450 nm by a SPECTRA max Microplate Reader (model 680, Bio-Rad, Tokyo, Japan), and the cell viability of each concentration was calculated according to the formula provided by the manufacturer. These experiments were repeated three times.

### Animals

Female BALB/c nude mice (4- to 6-week-old) were obtained from Xiamen University Laboratory Animal Center (Xiamen, China). The mice were reared in a sterile environment and maintained for a 12-h light/dark cycle. The animals were injected with HepG2 cells (1.0 × 10^7^/mL) subcutaneously to induce tumor formation. All of the animal experiments in this work were conducted according to the protocol approved by the Animal Care and Use Committee of Xiamen University.

### Biocompatibility of BiF_3_: Ln@PVP NPs In Vivo

Histological analysis was used to observe the biocompatibility of BiF_3_: Ln@PVP NPs in vivo. The experimental group mice were injected with a BiF_3_: Ln@PVP NP suspension at 200 mg/kg through the tail vein; control mice were intravenously injected with the same volume of PBS. After 24 h, the major organs including hearts, livers, spleens, lungs, kidneys, and brains were immediately removed after the mice were sacrificed. All of the organs were fixed with 4% paraformaldehyde fixative for 12 h and then embedded in paraffin and sliced. Finally, hematoxylin–eosin (H&E) staining was performed. The morphology of the organs was evaluated and captured by an upright fluorescence microscope (Leica DM2700 P, Germany).

### CT Performance of BiF_3_: Ln@PVP NPs In Vitro and In Vivo

To study the application of BiF_3_: Ln@PVP NPs in vitro CT imaging, BiF_3_: Ln@PVP NP and Iohexol suspensions were prepared and diluted to 0, 0.625, 1.25, 2.5, 5.0, 10.0, and 20.0 mg/mL and removed into 0.3-mL Eppendorf tubes. The CT images and the corresponding CT values of BiF_3_: Ln@PVP NP and Iohexol suspensions were obtained and recorded by an X-ray CT instrument (Siemens) with an operating voltage of 50 kV and 80 kV, respectively. Next, the CT imaging ability of BiF_3_: Ln@PVP NPs in vivo was studied; The BiF_3_: Ln@PVP NP suspension was intratumorally injected into the tumor-bearing nude mice at 200 mg/kg (100 μL). Subsequently, the mice were anesthetized and the X-ray CT machine (Siemens, 80 kV, 88 μA) was used to capture CT images before and after administration of BiF_3_: Ln@PVP.

### CT Performance of BiF_3_: Ln@PVP NPs in GI Tract and Histological Analysis

To further explore the value of BiF_3_: Ln@PVP NPs in CT imaging, the mice were fasted overnight and orally administrated a BiF_3_: Ln@PVP NP suspension (300 μL, 20 mg/mL) through a gastric tube. The mice were then intraperitoneally anesthetized with chloral hydrate. Next, GI images at different intervals (0, 15 min, 30 min, 120 min, 6 h, 12 h, 24 h, and 48 h) were captured at 80 kV. Finally, 3D models of the mice were reconstructed via the CT machine. The mice were then sacrificed and the stomachs, small intestines and large intestines were removed and fixed with 4% paraformaldehyde for 12 h. They were then embedded in paraffin and sectioned before H&E staining to evaluate the gastrointestinal toxicity of BiF_3_: Ln@PVP NPs.

### Statistical Analysis

Date were analyzed using one-way ANOVA; a *P* value < 0.05 was considered statistically significant in all analyses (95% confidence level).

## Results and Discussion

### Fabrication and Physicochemical Properties of the BiF_3_: Ln@PVP NPs

**Scheme 1.**
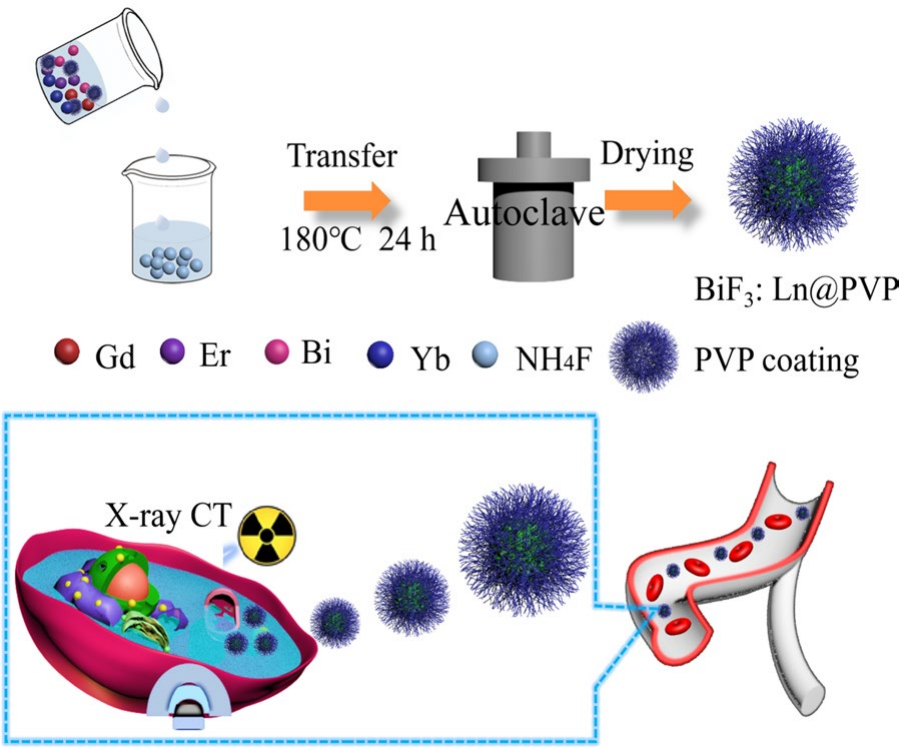
Schematic diagram of BiF_3_: Ln@PVP NPs synthesis process and its applications

**Fig. 1:**
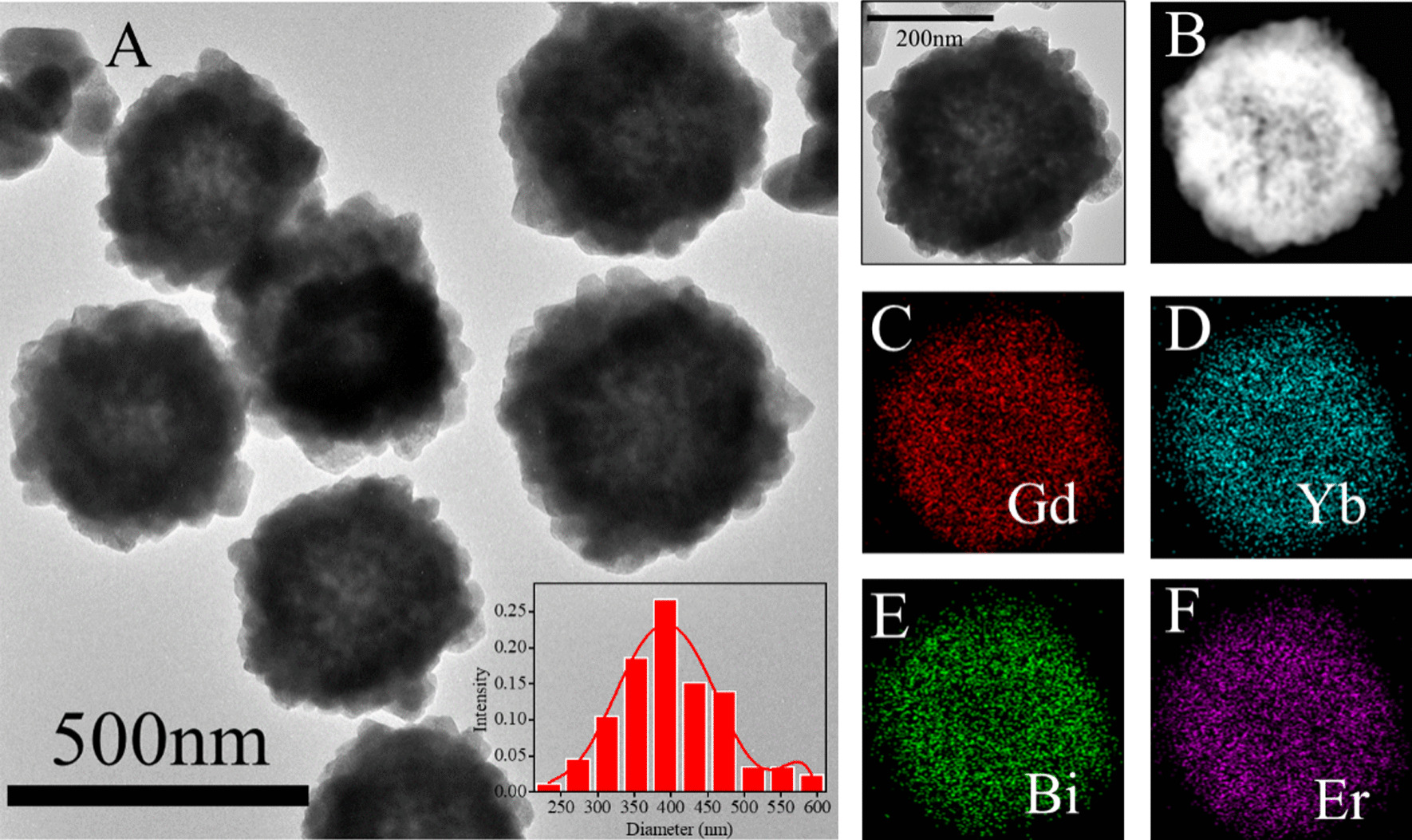
Morphology and particle size of the BiF_3:_ Ln@PVP NPs. **A** TEM images of the BiF_3:_ Ln@PVP NPs and its particle size distributions (bottom right). **B**–**F** Dark-field TEM of image of BiF_3:_ Ln@PVP NPs and corresponding TEM elemental maps of Gd, Yb, Bi and Er

First, the BiF_3_: Ln@PVP NPs were prepared through a hydrothermal reaction (Scheme 1). Figure 1A shows the morphology of the BiF_3_: Ln@PVP NPs by TEM. The BiF_3_: Ln@PVP NPs have a uniform and spherical structure. The mean size of the BiF_3_: Ln@PVP NPs is about 380 nm and is evenly dispersed. The insert figure shows that the nanoparticles have a relatively narrow particle size distribution (bottom right). The composition of BiF_3_: Ln@PVP NPs were analyzed by EDS after evaluating the morphology of the BiF_3_: Ln@PVP NPs. Figure 1B–F shows a dark-field image of BiF_3_: Ln@PVP NPs taken before elemental analysis. The results show that our nanoparticles are mainly composed of Gd, Yb, Er, and Bi elements, indicating that the BiF_3_: Ln@PVP NPs were successfully synthesized.

**Fig. 2:**
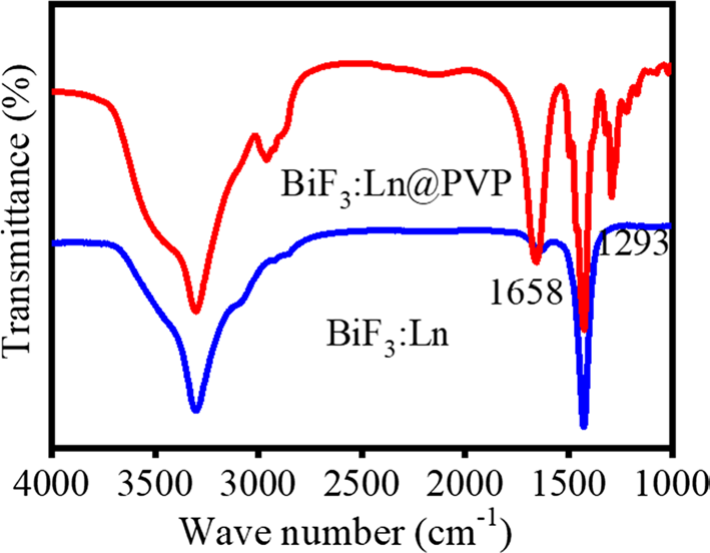
FTIR spectra of BiF_3:_ Ln@PVP NPs. The blue line represents the initial absorption peak of the BiF_3_: Ln. The red line represents the absorption peak after modifying PVP to the surface of nanoparticles

**Fig. 3:**
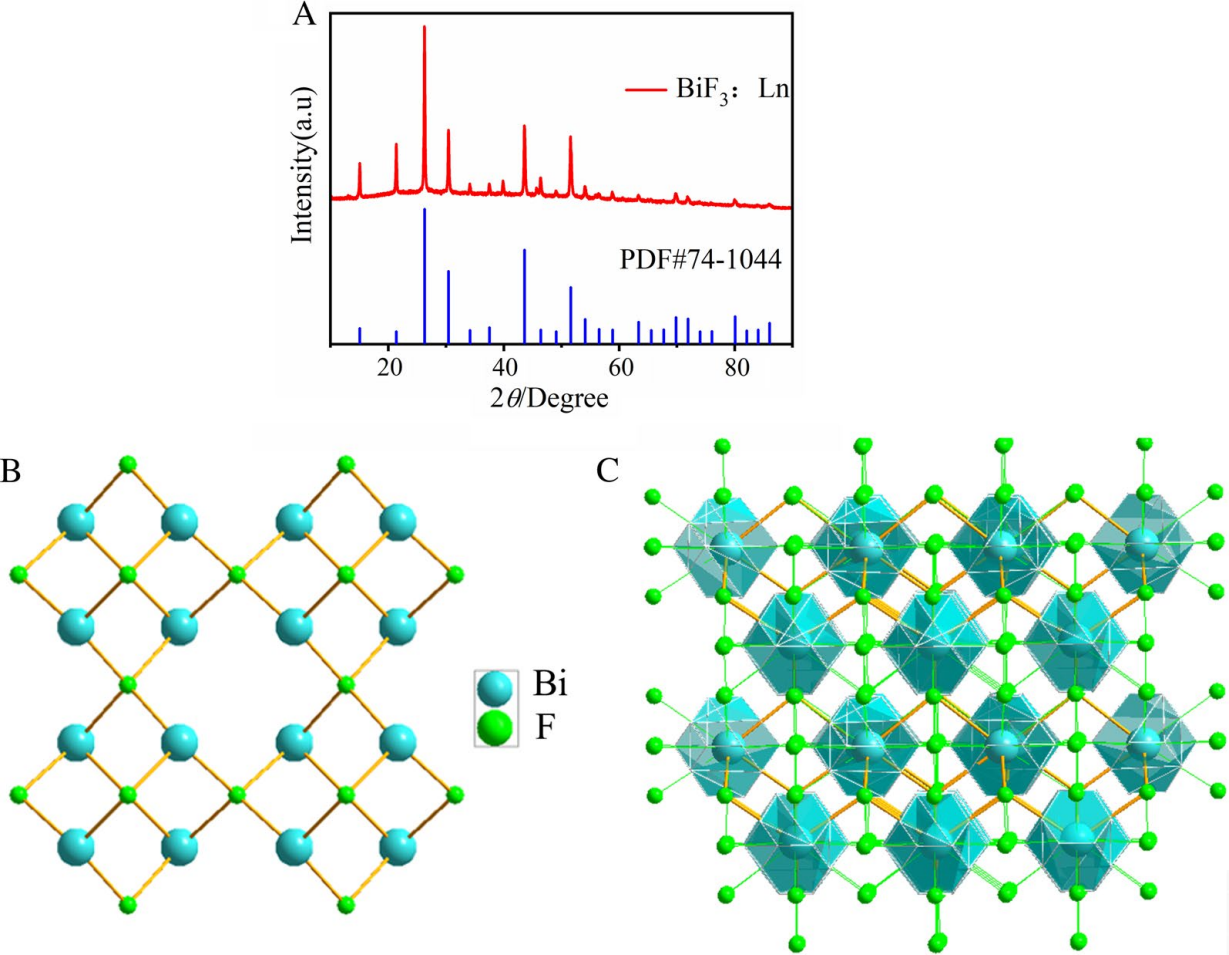
XRD pattern of BiF_3_: Ln@PVP NPs. **A** All peaks of the BiF_3_: Ln are well-matched with standard card BiF_3_: Ln data (PDF 74-0144). **B** The view of atomic distribution from C axis and **C** show the coordination along the A axis

**Fig. 4:**
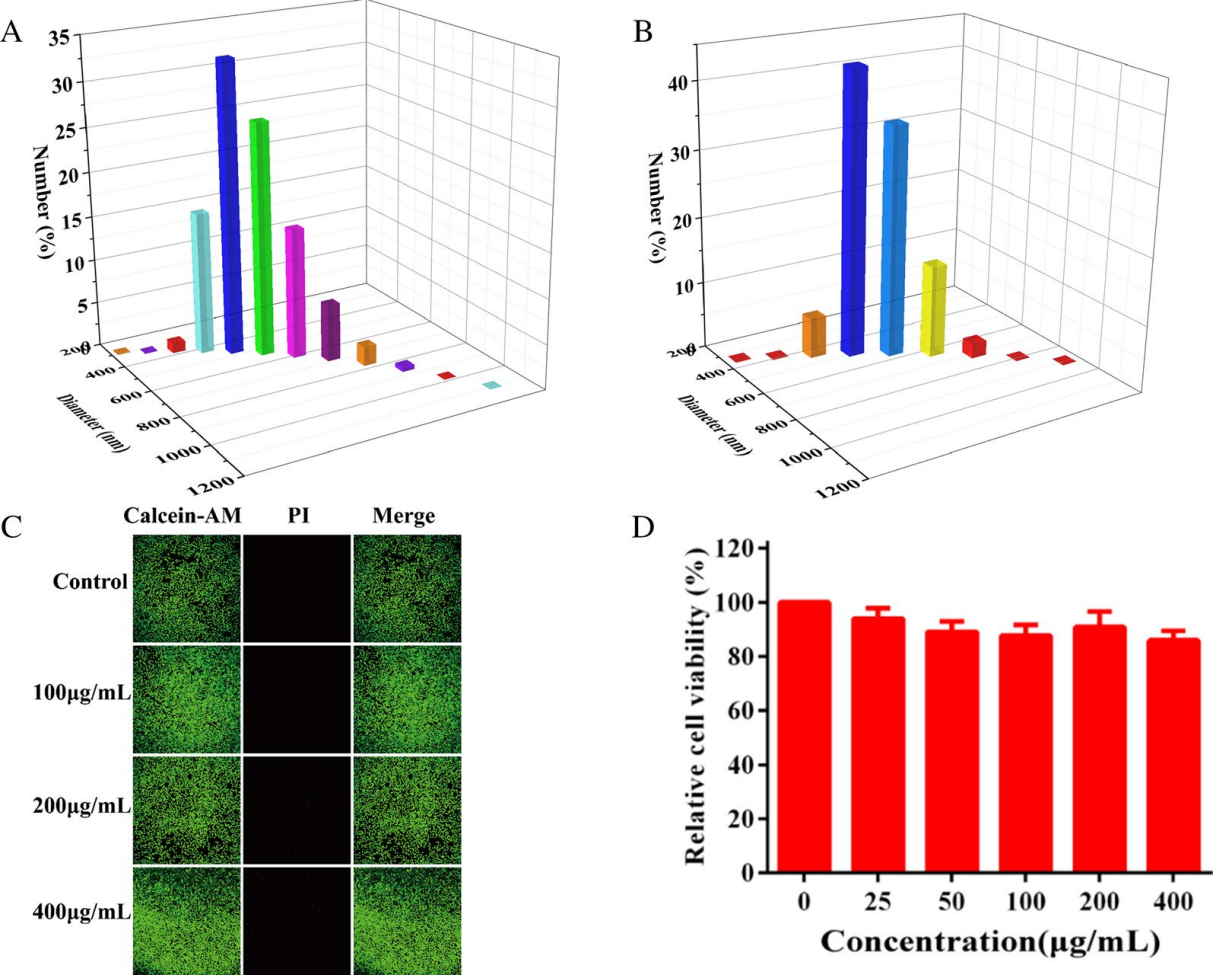
Stability and cytocompatibility of BiF_3_: Ln@PVP NPs. **A** The hydrodynamic diameter of BiF_3_: Ln@PVP NPs in DI and **B** PBS (pH 7.4). **C** Live-dead assay and **D** CCK-8 assay of the HepG2 cells treated with different concentrations of the BiF_3_: Ln@PVP NPs for 24 h

PVP is an effective stabilizer to improve the biocompatibility and stability of nanomaterials [32]. Therefore, we modified our nanoparticles with PVP as previously reported [33]. FTIR spectra were used to determine whether the PVP was successfully coated on the surface of the nanoparticles (Fig. 2). There were C=O group strong absorption peaks and C–N group peaks at 1658 and 1293 cm^−1^, respectively. These were from PVP indicating that the coating of PVP on the surface of the nanoparticles was complete [34]. The XRD pattern of the BiF_3_: Ln@PVP NPs are shown in Fig. 3. Figure 3A shows that all peaks are well-matched with standard card BiF_3:_ Ln data (PDF 74-0144) further demonstrating that the BiF_3_: Ln@PVP NPs were successfully prepared. The atomic parameters of the BiF_3_ structure can be used as the initial parameters in the standard cif card via Diamond software. The standard structure yielded PDF 74-0144, *a* = *b* = *c* = 5.865 Å, *V* = 201.75(3) Å, and density (*c*) = 8.755. The BiF_3_ crystal structure viewed from the C-axis has layers stacked in the direction perpendicular to the A-axis (Fig. 4B), and the view of a single structure from the A-axis shows Bi is in the center of the atom (Fig. 4C). These results indicate that BiF_3_: Ln@PVP NPs have a good crystal structure, and the surface coating only slightly influences the crystal structure of BiF_3_: Ln@PVP NPs.

### Stability and Cytocompatibility of BiF_3_: Ln@PVP NPs

Since the dispersion size of nanoparticles can affect the interaction with biological systems, it is necessary to study the dispersion size of nanoparticles in different solutions [33]. Figure 4A, B shows that the BiF_3:_ Ln@PVP NPs have a relatively narrow distribution in DI and PBS (pH = 7.4), indicating that the BiF_3:_ Ln@PVP NPs have good stability in different solutions. Thus, they are suitable for biological applications.

The cytotoxicity of BiF_3_: Ln@PVP NPs were studied after proving that BiF_3:_ Ln@PVP NPs have good stability in different solutions. The live-dead experiment evaluated the cytotoxicity of BiF_3:_ Ln@PVP NPs. No obvious red fluorescence was observed when the concentration of BiF_3_: Ln@PVP NP suspension reached 400 µg/mL and was cultured with HepG2 cells for 24 h (compared with the control group; Fig. 4C). This indicates that there was no significant cell death in the experimental group. Subsequently, a CCK-8 assay was performed to further study the cytotoxicity of BiF_3_: Ln@PVP NPs. Figure 4D shows the cell viability of HepG2 cells incubated with various concentrations of BiF_3_: Ln@PVP NP suspensions for 24 h, the experimental groups of HepG2 cells all had relatively high cell viabilities. Furthermore, the cell viability was as high as 85.96% when the concentration of the BiF_3:_ Ln@PVP NP suspension reached to 400 μg/mL. These results demonstrated that the BiF_3:_ Ln@PVP NPs possessed favorable biocompatibility in vitro, which may be attributed to the coating of PVP on the surface of the BiF_3_: Ln@PVP NPs.

### Biocompatibility of BiF_3_: Ln@PVP NPs In Vivo

**Fig. 5:**
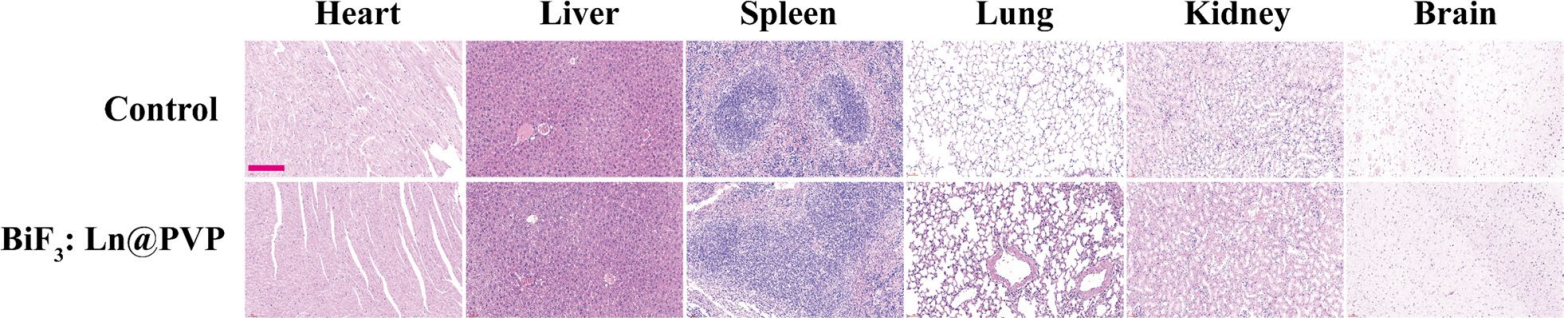
H&E staining images of major organs before and after BiF_3_: Ln@PVP NPs administration (scale bar 200 µm)

In addition to low cytotoxicity, good in vivo biocompatibility is another necessary condition for the clinical use of contrast agents [35]. Thus, the BiF_3:_ Ln@PVP NP suspension was prepared and injected into the mice at 200 mg/kg (100 μL) through the tail vein. The same volume of PBS solution was injected and set as the control group. After 24 h, the mice were sacrificed, and major organs were excised during necropsy. The H&E staining was performed to evaluate the system toxicity. Figure 5 shows no obvious pathological abnormalities after BiF_3_: Ln@PVP NPs administration for 24 h. These results indicate that BiF_3_: Ln@PVP NPs have good biocompatibility, which is consistent with the low cytotoxicity shown above.

### The Ability of BiF_3_: Ln@PVP NPs In Vitro CT Imaging

**Fig. 6:**
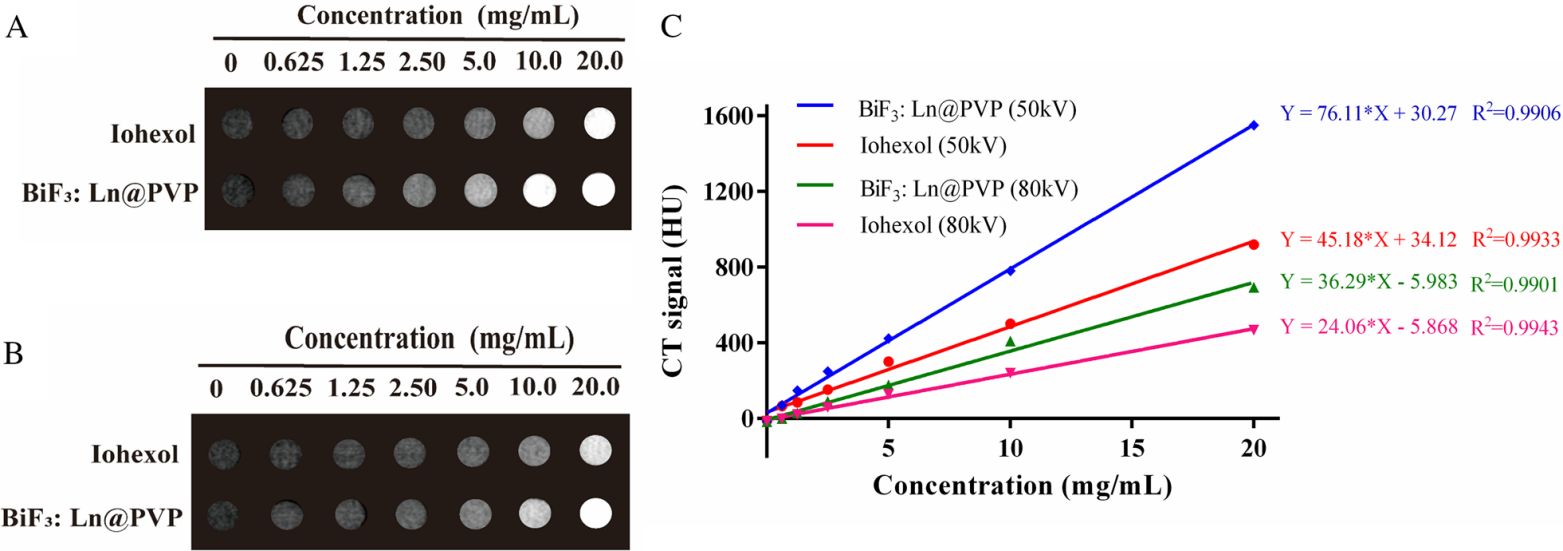
Comparison of the effect of in vitro CT imaging between BiF_3:_ Ln@PVP NPs and Iohexol. **A**, **B** In vitro CT imaging under different operating voltages (50 and 80 kV, respectively) of BiF_3:_ Ln@PVP NPs and Iohexol. **C** The corresponding CT values of BiF_3:_ Ln@PVP NPs and Iohexol under 50 and 80 kV, respectively

Elements with high atomic numbers usually have high contrast effects because of their great X-ray attenuation. For example, contrast agents prepared from precious metals with a high atomic number (Au [36], Ag [37], etc.) have excellent CT imaging effects as previously reported. Therefore, a promising type of contrast agent can be considered. However, their high cost limits their further clinical application. Bismuth has good biological safety and low cost, with great X-ray attenuation ability [38–41]. Herein, to evaluate the contrast agent effect of BiF_3_: Ln@PVP NPs, we compared the X-ray attenuation ability of BiF_3_: Ln@PVP NPs with the commercial contrast agent Iohexol solution in vitro. Figure 6A, B shows the corresponding CT images of BiF_3_: Ln@PVP and Iohexol under different operating voltage (50 kV and 80 kV, respectively). Figure 6A, B indicates that the gray level of the image gradually changes from black shade to white shade as the concentration of the suspensions increased. However, at the same concentration, BiF_3_: Ln@PVP has a brighter shade than Iohexol because the X-ray attenuation coefficient of Bi is higher I (Bi is 5.74 cm^2^ kg^−1^ and I is 1.94 cm^2^ kg^−1^ at 100 keV) [42].

Figure 6C shows that the CT value (Hounsfield Unit, HU) increases linearly with increasing BiF_3_: Ln@PVP NPs and Iohexol concentration (both *R*^2^ > 0.99) regardless of the operating voltage. The CT value of the unit mass concentration of BiF_3_: Ln@PVP NPs is much higher than that of Iohexol (1.5- and 1.7-fold higher than that at 50 kV and 80 kV, respectively). These results indicate that the BiF_3_: Ln@PVP NPs can provide better contrast effect at the same doses versus commercial Iohexol; these data confirm that the BiF_3_: Ln@PVP NPs have good in vitro CT imaging capability, which is of great significance because it can reduce the amount of contrast agent while ensuring good imaging effects. This can significantly reduce the toxicity and side effects.

### Contrast Effect of BiF_3_: Ln@PVP NPs In Vivo CT Imaging

**Fig. 7:**
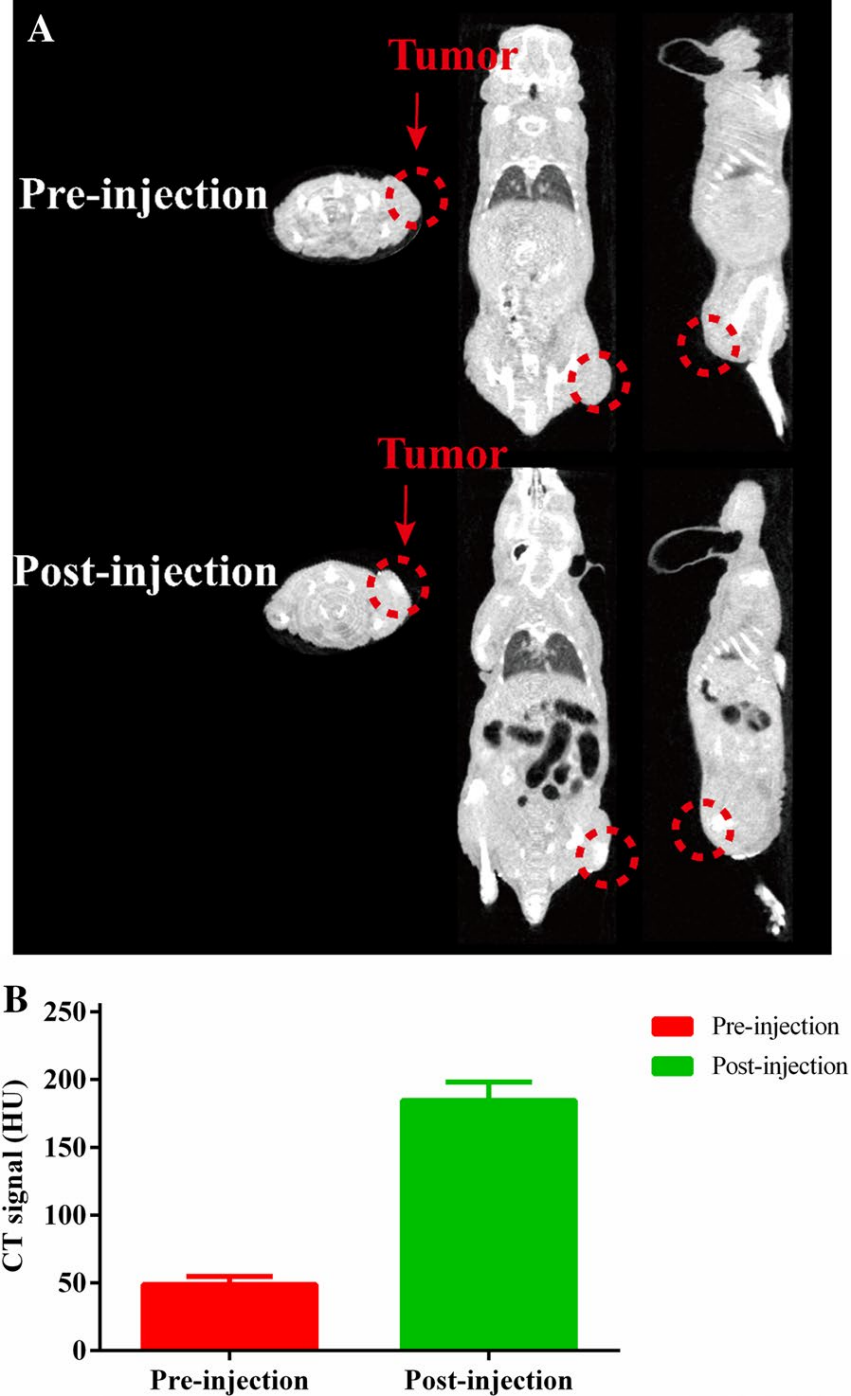
BiF_3:_ Ln@PVP NPs in vivo CT imaging effect. **A** CT images before and after BiF_3:_ Ln@PVP NPs injection and **B** the corresponding CT value. The red circle indicates tumor tissue

The BiF_3_: Ln@PVP NP suspension was next injected intratumorally into the tumor-bearing mice (200 mg/kg, 100 µL) to evaluate the contrast effect of BiF_3_: Ln@PVP NPs in vivo CT imaging. A strong signal intensity change is detected versus baseline in the same tumor area after 1 h administration of BiF_3_: Ln@PVP NP suspension (Fig. 7A). Meanwhile, Fig. 7B shows that the CT value of post-injection (184.58 HU) is much higher than pre-injection (48.9 HU). This is due to the increase in the X-ray attenuation coefficient of tumor tissue after BiF_3_: Ln@PVP NPs are distributed in tumor tissue. The results indicate that the BiF_3_: Ln@PVP NPs have great in vivo CT imaging ability.

### GI Tract CT Imaging Performance of BiF_3_: Ln@PVP NPs and Its GI Toxicity

Encouraged by the promising results above, we were motivated to further evaluate potential application of BiF_3_: Ln@PVP NPs in CT imaging. As a common noninvasive imaging method, CT plays a vital role in the diagnosis of GI diseases and the formulation of treatment plans due to its convenient image processing, no tissue damage, and painlessness of patients [43, 44]. The commonly used barium sulfate contrast agent is usually used together with aerogenic powder. Owing to the different densities produced by the two substances, the GI tract cannot always be clearly displayed resulting in missed diagnoses, which limit its in clinical use [45]. Thus, it is of great significance to explore high-efficiency GI contrast agents that do not require additional assistance. In this work, we explored the effect of BiF_3_: Ln@PVP NPs on the GI tract in nude mice.

**Fig. 8:**
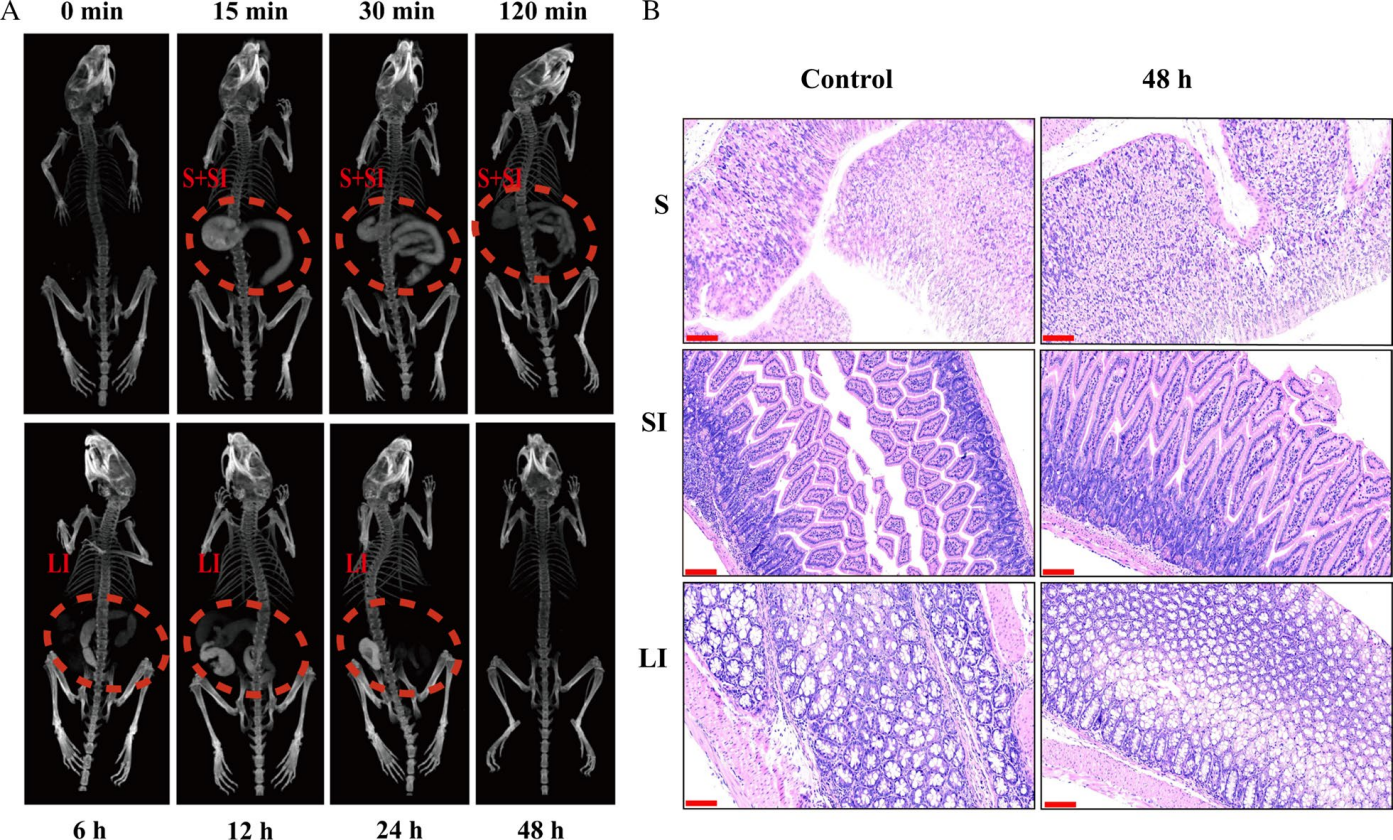
**A** CT images of the GI tract after oral administration of BiF_3:_ Ln@PVP NPs at different intervals (0, 15 min, 30 min, 120 min, 6 h, 12 h, 24 h, and 48 h). **B** H&E staining images of stomach, small intestine, and large intestine before and after oral administration of BiF3: Ln@PVP NPs (scale bar, 200 µm). “S,” “SI,” and “LI” represent stomach, small intestine, and large intestine, respectively

Figure 8A shows that the shape of the stomach and small intestine became visible after oral administration of BiF_3_: Ln@PVP NP suspension (20 mg/mL, 300 μL) for 15 min. At 30 min, the BiF_3_: Ln@PVP NPs were metabolized with the peristalsis of the stomach. The stomach morphology became weaken. At 120 min, most of the BiF_3_: Ln@PVP NPs were metabolized from the stomach, and only the remaining stomach outline was visible. The BiF_3_: Ln@PVP NPs began to appear in the contour of the large intestine at 6 h, indicating that the nanoparticles began to become enriched in the large intestine; the morphology of the large intestine was clearly visible at 12 h. Most of the BiF_3_: Ln@PVP NPs were excreted and only a small part remains at 24 h. We could not observe the GI morphology at 48 h interval, indicating that all the BiF_3_: Ln@PVP NPs were excreted from GI tract. After the nanoparticles were completely excreted from the GI tract, the mice were sacrificed and the stomach, small intestine and large intestine were removed for an H&E assay to evaluate the GI toxicity of the BiF_3_: Ln@PVP NPs. Figure 8B shows no obvious histological changes in the stomach, small intestine, or large intestine after 48 h of oral administration of BiF_3_: Ln@PVP NPs demonstrating that the BiF3: Ln@PVP NPs have no significant toxicity to the GI tract. These results indicate that BiF_3_: Ln@PVP NPs can be used as a potential CT contrast agent for the GI tract to enhance the CT imaging performance of the GI tract, while having no obvious toxicity to the GI tract.

These results indicate that BiF_3_: Ln@PVP NPs have potential as clinical CT contrast agents for tumor and gastrointestinal imaging. However, owing to the limitation of particle size, BiF_3_: Ln@PVP NPs cannot achieve good enhanced permeability and retention (EPR) effect [46]. The long-term biological safety of BiF_3_: Ln@PVP NPs and the metabolic process in vivo requires further study.

## Conclusion

Herein, we synthesized a novel CT contrast agent via hydrothermal process. The TEM data show that BiF_3_: Ln@PVP NPs have a uniform spherical structure with a mean size of about 380 nm. The FTIR spectra show that the PVP was successfully wrapped on the surface of the nanoparticles to improve the biological safety of the nanoparticles. We then compared the in vitro CT imaging effect with Iohexol under different operating voltages. The results indicate that the BiF_3_: Ln@PVP NPs have a better X-ray attenuation ability than Iohexol. Biocompatibility studies show that the BiF_3_: Ln@PVP NPs have no obvious toxicity to major organs in vivo. Finally, the good X-ray attenuation ability allows BiF_3_: Ln@PVP NPs to have good contrast imaging effects in vivo to successfully visualize the GI tract in detail without causing damage to the GI tract. Therefore, our work offers a high-efficiency CT contrast agent with good water-soluble stability, good biosafety, and high efficiency. These features make it a potential candidate for clinical contrast agents.

## Data Availability

The datasets used and/or analyzed during the current study are available from the corresponding author on reasonable request.

## Abbreviations

CT: Computed tomography
PVP: Polyvinylpyrrolidone
GI: Gastrointestinal
PLAL: Pulsed laser ablation in liquid
Bi: Bismuth
Gd: Gadoliniumnitrate
Yb: Ytterbium
Er: Erbium
Au: Gold
Ta: Tantalum
Pt: Platinum
I: Iodine
NP: Nanoparticle
CCK-8: Cell counting kit-8
RPMI: Roswell Park Memorial Institute
FBS: Fetal bovine serum
Ln: Lanthanides
DI: Deionized water
TEM: Transmission electron microscopy
EDS: Energy dispersive spectrum
FTIR: Fourier transform infrared spectroscopy
XRD: Powder X-ray diffraction
DLS: Dynamic light scattering
PI: Propidium iodide
H&E: Hematoxylin–eosin
HU: Hounsfield unit
EPR: Enhanced permeability and retention
